# The Dual Role of an ESCRT-0 Component HGS in HBV Transcription and Naked Capsid Secretion

**DOI:** 10.1371/journal.ppat.1005123

**Published:** 2015-10-02

**Authors:** Shu-Fan Chou, Ming-Lin Tsai, Jyun-Yuan Huang, Ya-Shu Chang, Chiaho Shih

**Affiliations:** 1 Graduate Institute of Microbiology, College of Medicine, National Taiwan University, Taipei, Taiwan; 2 Institute of Biomedical Sciences, Academia Sinica, Taipei, Taiwan; 3 Graduate Institute of Life Sciences, National Defense Medical Center, Taipei, Taiwan; University of California, San Diego, UNITED STATES

## Abstract

The Endosomal Sorting Complex Required for Transport (ESCRT) is an important cellular machinery for the sorting and trafficking of ubiquitinated cargos. It is also known that ESCRT is required for the egress of a number of viruses. To investigate the relationship between ESCRT and hepatitis B virus (HBV), we conducted an siRNA screening of ESCRT components for their potential effect on HBV replication and virion release. We identified a number of ESCRT factors required for HBV replication, and focused our study here on HGS (HRS, hepatocyte growth factor-regulated tyrosine kinase substrate) in the ESCRT-0 complex. Aberrant levels of HGS suppressed HBV transcription, replication and virion secretion. Hydrodynamic delivery of HGS in a mouse model significantly suppressed viral replication in the liver and virion secretion in the serum. Surprisingly, overexpression of HGS stimulated the release of HBV naked capsids, irrespective of their viral RNA, DNA, or empty contents. Mutant core protein (HBc 1–147) containing no arginine-rich domain (ARD) failed to secrete empty virions with or without HGS. In contrast, empty naked capsids of HBc 1–147 could still be promoted for secretion by HGS. HGS exerted a strong positive effect on the secretion of naked capsids, at the expense of a reduced level of virions. The association between HGS and HBc appears to be ubiquitin-independent. Furthermore, HBc is preferentially co-localized with HGS near the cell periphery, instead of near the punctate endosomes in the cytoplasm. In summary, our work demonstrated the importance of an optimum level of HGS in HBV propagation. In addition to an effect on HBV transcription, HGS can diminish the pool size of intracellular nucleocapsids with ongoing genome maturation, probably in part by promoting the secretion of naked capsids. The secretion routes of HBV virions and naked capsids can be clearly distinguished based on the pleiotropic effect of HGS involved in the ESCRT-0 complex.

## Author Summary

Hepatitis B virus (HBV) is an important human pathogen. Chronic infection with HBV can lead to cirrhosis and liver cancer. While HBV infection is treatable, it remains a challenge to eradicate the virus in patients. HBV can produce various particles with different sizes and morphologies. Virions are complete particles with both nucleocapsids and surface antigen (HBsAg) envelope. In addition to DNA-containing virions, HBV produced incomplete particles, including HBsAg particles without nucleocapsids, naked capsid particles without the HBsAg envelope, and empty virions containing both HBsAg envelope and empty capsids. It remains unclear how these various HBV particles are exported from the liver. We identified many host factors known to be involved in a membrane trafficking machinery (so-called ESCRT), as well as required for HBV replication. Interestingly, aberrant expression of HGS (an ESCRT-0 factor) can inhibit HBV replication. However, unexpectedly, HGS could boost the release of naked capsids, while concurrently reduced the extracellular virions and HBsAg particles. HGS can associate and co-localize with HBV capsid protein. In summary, our work demonstrated that an appropriate level of HGS is important for HBV propagation. We entertain the possibility that HGS and other ESCRT factors may be further developed into a therapeutic treatment for hepatitis B.

## Introduction

Hepatitis B virus is a leading cause of chronic liver diseases and more than 240 million people are infected worldwide. Persistent infection with HBV can cause severe liver injury, cirrhosis, and the development of hepatocellular carcinoma (HCC) [1]. Though the HBV vaccination program has been successful in recent decades worldwide, the therapeutic efficacy of HBV remains limited [2,3]. The machinery of Endosomal Sorting Complex Required for Transport (ESCRT) was first identified in yeast [4]. One of the best-known functions of ESCRT is its role in targeting ubiquitinated cargos of recycled host factors into vacuoles, such as multivesicular bodies (MVBs) [5]. This ESCRT machinery can be divided into five components known as ESCRT-0, -I, -II, -III and VPS4 ATPase complex. ESCRT-0, I, and II can function in cargo recognition and sorting [6–8], along with the formation of membrane invagination [9]. The late-acting ESCRT-III is able to catalyse the scission of membrane necks, which can support the formation of intraluminal vesicles [10,11]. These sequentially formatted ESCRT complexes will be disassembled by VPS4-mediated ATPase activity and finally recycled back to the cytoplasm [12]. Many enveloped viruses, including human immunodeficiency virus (HIV), can coopt the ESCRT pathway during virion maturation and release [13]. Depletion of ESCRT proteins by either siRNA or dominant-negative mutants, both impaired membrane fission and blocked HIV virion release [14,15]. Previously, our laboratory investigated the potential role of an ESCRT factor VPS4 in HBV life cycle [16]. In addition to its involvement in secretion, surprisingly, VPS4 could have another role in intracellular HBV replication. Dominant-negative (DN) VPS4 mutants not only reduced HBV secretion in a dose-dependent manner, but also suppressed intracellular viral replication. Like the DN mutants of VPS4, overexpression of the wild type version of VPS4 also suppressed HBV replication in both HepG2 and HuH-7 cells [16]. Subsequent reports further supported that VPS4 is indeed important for HBV virion morphogenesis [17,18]. While ESCRT-I seemed to have no significant effect on HBV production, ESCRT-II and -III appeared to be involved in viral maturation and egress [18,19]. To date, it remains to be elucidated whether ESCRT-0 has a role in HBV life cycle.

When HBV nucleocapsids reach genome maturation in the cytoplasm, two major outcomes will follow. One is to undergo envelopment and achieve virion release facilitated by the ESCRT machinery, the other is to shuttle their mature genomes back to the nucleus for further amplification of the covalently closed circular DNA (ccc DNA) [20]. In addition to the infectious 42 nm virions as described above, hepadnaviruses in hepatocyte culture can secrete at least three other kinds of subviral particles, including 1) the 22 nm HBsAg particles; 2) the naked capsids without HBsAg envelope, approximately 28 nm in diameter [21], and 3) 42 nm empty virions [22–27]. HBsAg particles mainly assemble at the ER-Golgi Intermediate Compartment (ERGIC) and egress from the cells through the constitutive secretion pathway [28,29]. The secretion of naked capsids was reported to involve Rab33B [30] and ALIX (apoptosis linked gene II interacting protein X) in an ESCRT-independent manner [31]. Further investigation on the secretory mechanisms of these HBV viral and subviral particles could shed light on both basic science and translational medicine.

Here, we aim to decipher the potential relationships among ESCRT factors, HBV replication and egress. From our preliminary screening, we identified the cascade initiator ESCRT-0 complex as a critical component required for HBV replication. ESCRT-0 is known to consist of three cellular factors HGS, STAM1 and STAM2. In this study, perturbation of the stoichiometry of HGS in the ESCRT-0 complex could reduce viral gene expression, DNA replication, and virion release. Unlike the treatment with HGS specific siRNA, overexpression of HGS could significantly boost the secretion of naked capsids, at the expense of the reduced pool size of intracellular capsids, leading to diminished virion secretion. We speculate here that the egress routes of virions and naked capsids shared the common origin of precursors, and along the way, they branched out from each other. This conclusion is in part based on their differential responses to the pleiotropic effect of HGS in ESCRT-0, and in part based on their differential requirements of the arginine-rich domain (ARD) for egress.

## Results

### Screening and identification of ESCRT factors required for HBV replication

**Fig 1:**
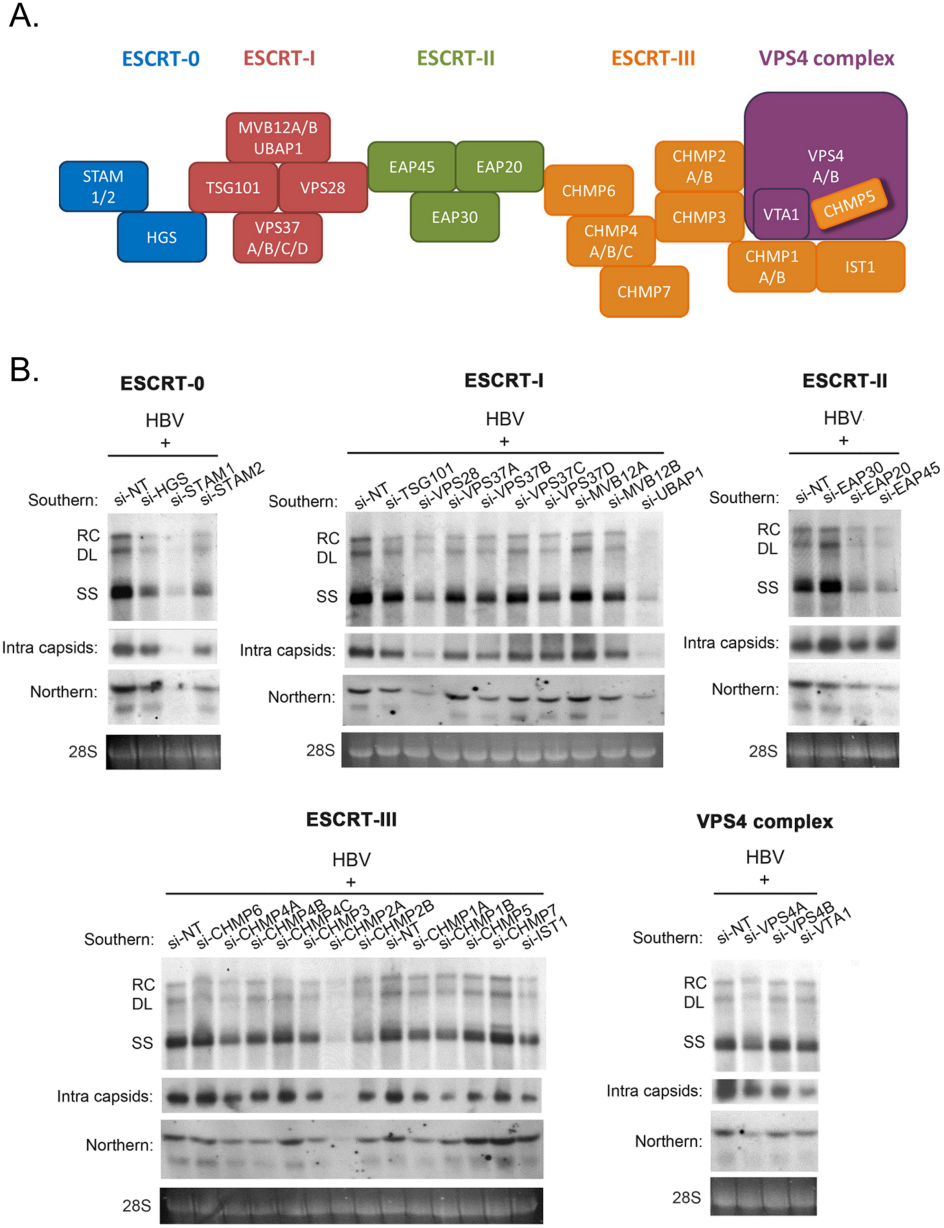
siRNA-knockdown screening for ESCRT factors required for HBV replication in HepG2 cells. (A) A cartoon illustrates the ESCRT cascade and the interactions between each component members of ESCRT-0, I, II, III, and the VPS4 ATPase complex. Of note, the exact stoichiometry of certain component members remains tentative [5]. (B) An HBV replicon plasmid containing a genomic dimer and individual siRNA against each ESCRT factor were co-transfected into HepG2 cells. Viral DNA replication, viral RNA expression and intracellular capsid particles were analyzed by Southern, Northern and Western blot analysis at day 5 post-transfection. The 28S rRNA was included as an RNA loading control. RC: relaxed circular DNA, DL: double-strand linear DNA, SS: single-strand DNA. We detected significant effect on HBV replication from 18 out of 30 tested ESCRT factors (as summarized in Table 1). Data shown here are representative of at least three independent experiments.

To investigate the potential role of the ESCRT machinery in the life cycle of HBV, we tested individually 30 different protein factors in the ESCRT complex by siRNA-knockdown screening (Fig 1A). Initially, an HBV replicon plasmid containing a genomic dimer (ayw) and pooled siRNAs specific for each ESCRT component, were co-transfected into HepG2 hepatoma cells. Five days post-transfection, we performed several assays for the transfected culture, including HBV DNA replication, RNA expression and intracellular capsid formation (Fig 1B). There was no apparent cytotoxicity upon siRNA treatment as measured by the MTT assay (S1A Fig). The respective knockdown efficiency of each siRNA against each host factor was validated by the real-time PCR (S1B Fig). Previously, we reported that DN VPS4 suppressed HBV replication [16]. Depletion of VPS4A by specific siRNA treatment was included as a positive control, which reduced intracellular HBV DNA replication and RNA expression in HepG2 cells (Fig 1B). Similar to VPS4A, we identified a number of ESCRT factors in the siRNA co-transfection screening, which significantly suppressed HBV DNA replication to various extents. These ESCRT factors include HGS, STAM1, and STAM2 of ESCRT-0, VPS28, VPS37B, and UBAP1 of ESCRT-I, EAP20 and EAP45 of ESCRT-II, CHMP4A, CHMP4B, CHMP3, CHMP2A, CHMP2B, CHMP1A, CHMP1B, and IST1 of ESCRT-III, as well as VTA1 of the VPS4 complex (Fig 1B and Table 1). In general, the suppression of viral DNA replication by these various ESCRT factors is associated with the reduced expression of HBV RNA and capsid particles by Northern and Western blot analyses, respectively (Fig 1B). Since many ESCRT factors (approximately 50%) did not have any effect on viral replication by the siRNA treatments, we speculate here that HBV replication does not require an intact or the entire ESCRT machinery (see Discussion).

**Table 1:** A list of tested ESCRT factors that affected HBV replication in HepG2 cells.

Complex	Suppression of HBV DNA replication
ESCRT-0	si-HGS
	si-STAM1, 2
ESCRT-I	si-VPS28
	si-VPS37B*
	si-UBAP1
ESCRT-II	si-EAP20
	si-EAP45
ESCRT-III & related factors	si-CHMP4A, B*
	si-CHMP3
	si-CHMP2A, B
	si-CHMP1A, B
	si-IST1
VPS4 complex	si-VPS4A
	si-VTA1*

* The effects of VPS37B, CHMP4B and VTA1 on HBV replication are modest, yet reproducible.

### ESCRT-0 complex is required for HBV replication

**Fig 2:**
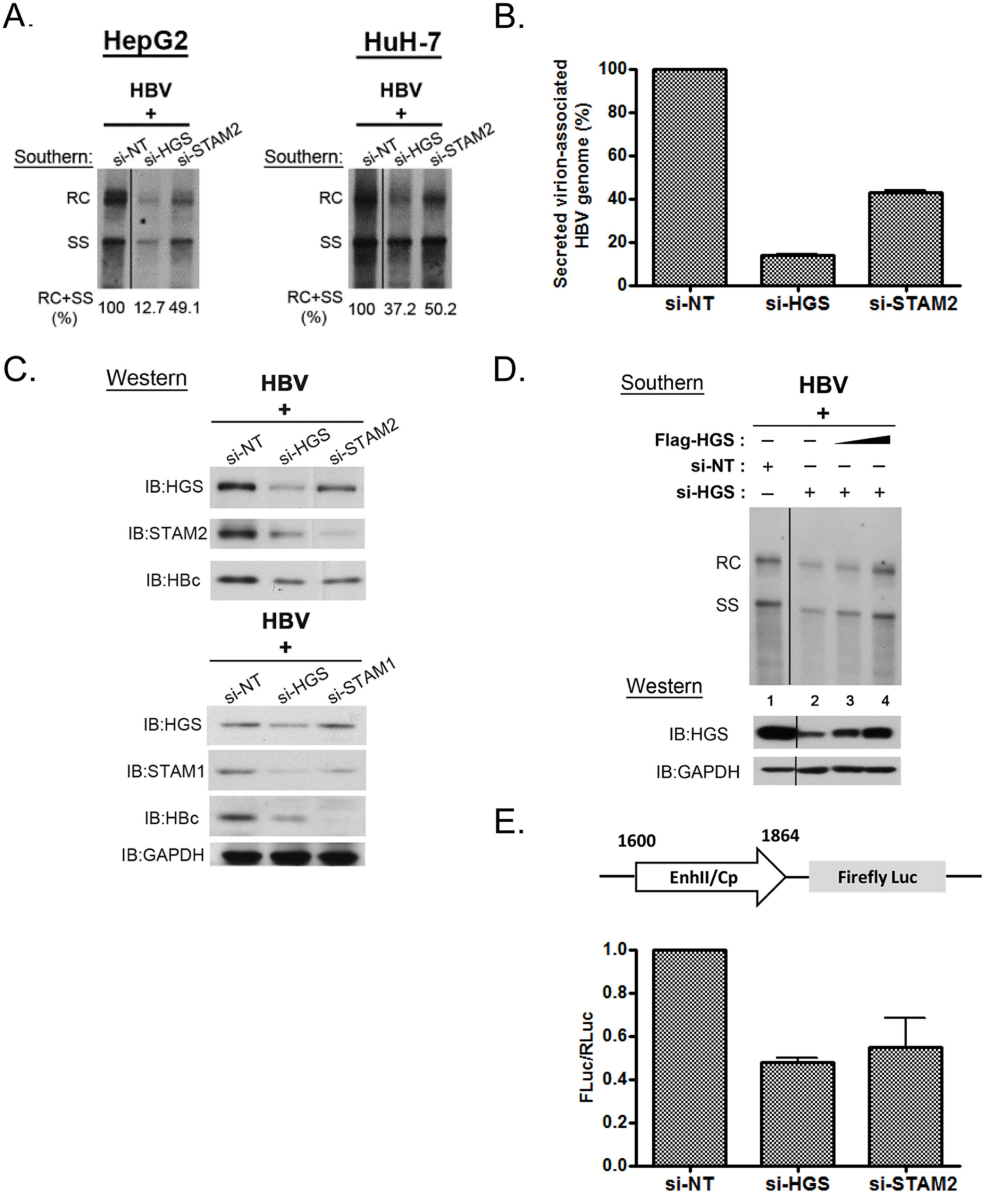
Knockdown of HGS destabilized ESCRT-0 complex and inhibited core protein expression and HBV replication. (A) Suppression of viral DNA replication was detected by Southern blot analysis in HepG2 and HuH-7 cells transfected with an HBV genomic dimer and siRNAs specific for HGS or STAM2. Signal intensity from RC and SS bandings was quantified by Image J software. (B) Significant reduction of virion-associated viral DNA was detected by qPCR in HepG2 cells upon depletion of ESCRT-0 components. Relative levels of HBV DNA in samples treated with si-HGS or si-STAM2 were normalized to that of the control sample treated with non-targeting siRNA (si-NT). Data here are representative of three independent experiments. (C) A co-depletion phenomenon was observed in HGS-depleted ESCRT-0 complex by SDS-PAGE analysis. Knockdown of HGS resulted in the concurrent reductions of STAM1, STAM2 and HBc protein expressions when the ESCRT-0 complex was destabilized. (D) The siRNA rescue experiment was performed by co-transfecting a constant amount of HBV replicon DNA with different doses of HGS expression vector (up to 0.2 μg per well in 6-well plate) in si-HGS (50 nM) treated HepG2 cells. Complementation of HGS efficiently restored the HGS protein level in a dose-dependent manner (lanes 2–4), as well as HBV replication. (E) Knockdown of ESCRT-0 factors inhibited HBV enhancer II and core promoter activity. A mixture of the reporter plasmid pGL3-EnhII-Cp, a control plasmid pRL-TK, and 50 nM siRNA, were co-transfected into HepG2 cells. Three days post-transfection, cell lysates were analysed by Dual-Glo Luciferase Assay System. The firefly luciferase activity driven by the HBV EnhII-Cp was always normalized first to an internal control of the renilla luciferase activity. The value of the Y-axis represents the relative luciferase activity in each siRNA-treated sample over the control siRNA-treated sample. Data are representative of three independent experiments.

Using the siRNA co-transfection assay, we found that the depletion of endogenous ESCRT-0 factors HGS, STAM1 and STAM2 significantly suppressed core-associated viral DNA replication, as compared to that of the non-targeting siRNA control (Figs 1B and 2A). In general, the respective inhibitory effects on HBV replication and core particle expression in HepG2 cells displayed an overall trend of si-STAM1 > si-HGS ≥ si-STAM2. A similar phenomenon was observed in another human hepatoma cell line HuH-7, albeit the inhibition was not as strong as that in HepG2 cells (Fig 2A). These results suggest that the ESCRT-0 complex *per se* was required for HBV DNA replication. Next, we measured the amount of virion-associated viral DNA in the supernatant of the co-transfected culture by quantitative PCR analysis. Depletion of endogenous HGS and STAM2 by siRNA treatments led to a substantial decline of viral DNA in the medium (Fig 2B). Taken together, we proposed that knockdown of the ESCRT-0 complex suppressed HBV intracellular replication, thereby resulting in the reduced virion production. By Western blot analysis, we noted that the knockdown of HGS significantly and simultaneously suppressed both STAM1 and STAM2 protein levels (Fig 2C). This result is reminiscent of a so-called “co-depletion” phenomenon [19,32], i.e., when the proper stoichiometry of each component in a complex is distorted, the stability of the whole complex and its associated components can be disturbed. Moreover, we noted that the co-depletion phenomenon of the ESCRT-0 complex is not necessarily reciprocal between HGS and STAMs. While depletion of HGS can cause concurrent reduction of STAM1 and STAM2, the converse was not observed (Fig 2C). This non-reciprocal co-depletion phenomenon can perhaps be explained by the functional redundancy between STAM1 and STAM2 proteins [33]. Similarly, a previous report had demonstrated that HGS could control the degradation of STAMs protein [34], which implied a critical role of HGS in maintaining the integrity of the ESCRT-0 complex and its component factors. Consistent with the reduction of intracellular capsids (Fig 1B), when the ESCRT-0 complex was destabilized by the siRNA treatment, the HBc protein level was also reduced by SDS-PAGE analysis (Fig 2C). To rule out the possibility of any off-target effect from the HGS siRNA, we performed a rescue experiment by co-transfecting an increasing amount of HGS expression vector DNA in HepG2 cells treated with a constant amount of si-HGS. Indeed, complementation of HGS efficiently restored the HGS protein level and viral replication in a dose-dependent manner (compare lanes 2–4, Fig 2D).

Since depletion of ESCRT-0 reduced both viral RNA and protein expression, we further examined whether ESCRT-0 had any effect on the viral RNA transcription by performing a luciferase reporter assay. The firefly luciferase activity driven by the HBV enhancer-II/core promoter (EnhII-Cp) was always normalized to an internal control plasmid pRL-TK, which contains the renilla luciferase activity driven by the thymidine kinase (TK) promoter. The relative luciferase activities were reduced in treatments with either si-HGS or si-STAM2 (Fig 2E). This result suggests that the depletion of ESCRT-0 can suppress HBV-specific EnhII-Cp activity, and thus inhibited HBV transcription and replication. In addition, we detected no appreciable effect of si-HGS on the reporter activity driven by a TK-promoter (S2A Fig), or on viral replication driven by a CMV promoter (S2B Fig). Similarly, we detected no si-HGS effect on the expression of cellular housekeeping genes, e.g., GAPDH (Fig 2C). Taken together, it is tempting to speculate that the effect of si-HGS on viral replication is from the specific transcriptional suppression of HBV EnhII-Cp, rather than from a general or global effect on HepG2 cells.

### Aberrant stoichiometry of HGS suppressed HBV transcription and replication

**Fig 3:**
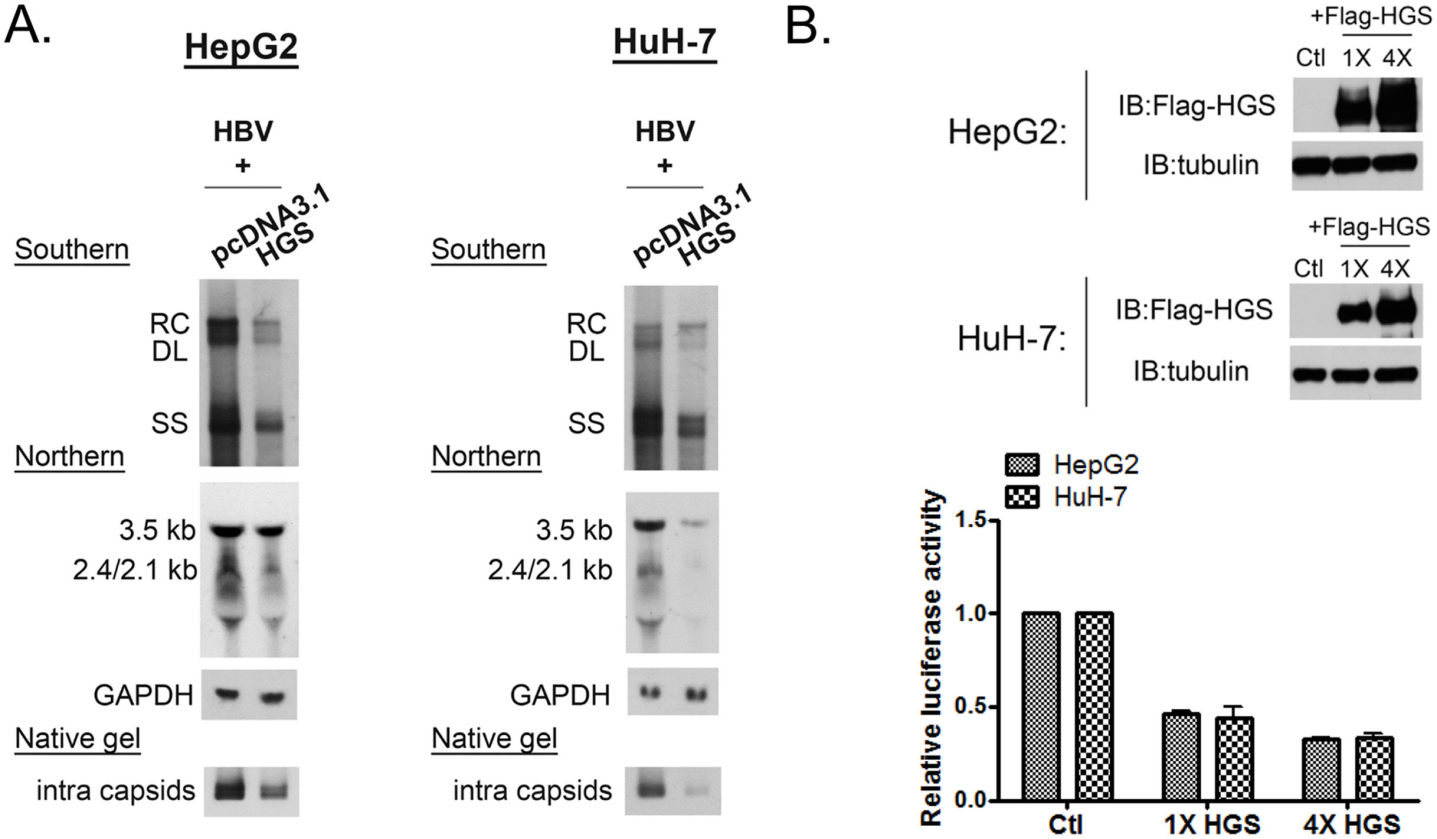
Overexpressed HGS suppressed HBV transcription and replication. (A) Overexpression of HGS reduced HBV DNA, RNA, and capsid particles. Plasmid DNAs of an HBV replicon pCHT-9/3091 and an HGS expression vector were co-transfected into HepG2 and HuH-7 cells (2:1 ratio). Intracellular core particle-associated viral DNA, RNA and capsid particles were examined at day 5 post-transfection. (B) Overexpression of HGS inhibited HBV EnhII-Cp activity. The reporter plasmid of pGL3-EnhII-Cp (as depicted in Fig 2E), control plasmid pRL-TK, and two doses of HGS expression vector (1X: 25 ng, 4X: 100 ng), were co-transfected into HepG2 and HuH-7 cells. Three days post-transfection, cell lysates were subjected to Western blot and reporter analysis. Increasing doses of the HGS protein further suppressed HBV EnhII-Cp activity.

Intriguingly, we observed that the overexpressed wild type HGS protein can also suppress viral DNA synthesis (Fig 3A). In addition to the reduction in DNA replication, viral RNA and capsid particles were both reduced upon HGS overexpression in HepG2 and HuH-7 cells. This is similar to the VPS4 story that we had reported earlier [16], both knockdown and overexpression of ESCRT-0 factor HGS could be harmful to viral replication. Consistent with the reduced viral RNA level, when Flag-HGS was overexpressed in a dose-dependent manner (Fig 3B, upper panel), gradual reduction in HBV EnhII-Cp activity was observed (Fig 3B, lower panel). Taken together, improper stoichiometry of the HGS protein in the ESCRT-0 complex appeared to result in significantly reduced viral RNA transcription and DNA replication. Of note, overexpression and depletion of the endogenous HGS appeared to have a difference in promoter specificity. Overexpressed HGS cannot discriminate between CMV promoter in pCHT-9/3091 (Fig 3A) and native promoter in HBV tandem dimer (S3 Fig) in gene expression and viral replication. However, depletion of endogenous HGS by siRNA exhibited a stronger suppression effect on native HBV promoter (Fig 2E and S2 Fig).

### Hydrodynamic delivery of HGS significantly suppressed HBV replication *in vivo*

**Fig 4:**
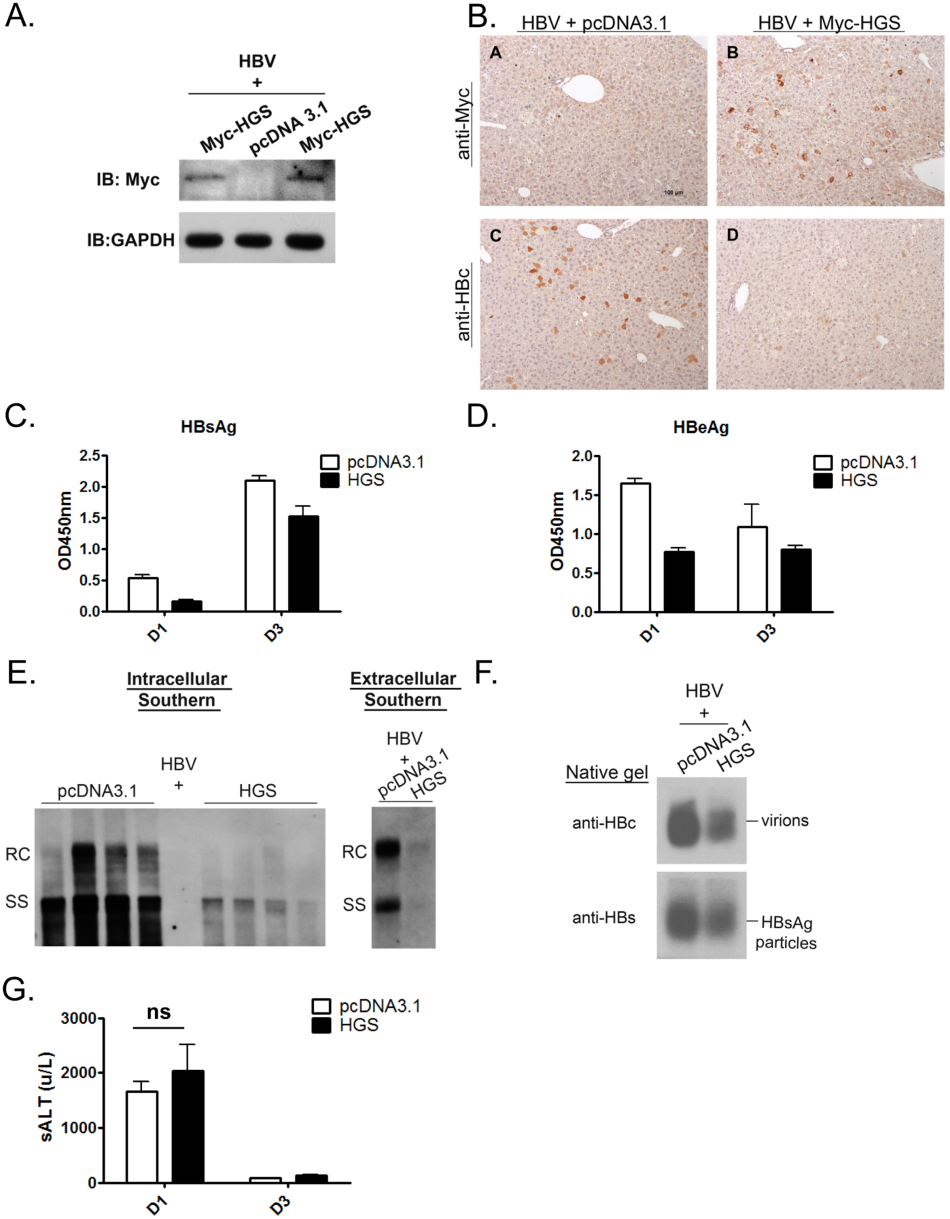
Overexpressed HGS significantly suppressed HBV replication *in vivo*. (A) The expression of HGS protein can be detected in the liver sample of hydrodynamically injected mice at 1 dpi by Western blot analysis. Six to eight week old BALB/c mice were tail-vein injected with 14 μg plasmid DNA of an HBV replicon (adr, dimer) and 6 μg DNA of an HGS expression vector or a control plasmid. (B) Immunohistochemistry analysis detected less HBc protein in sectioned liver in the presence of Myc-HGS at 1 dpi. (C) Secreted HBsAg (1000X dilution) and (D) HBeAg (10X dilution) in mouse sera were reduced upon HGS co-injection at 1 and 3 dpi by ELISA. Data are representative of three independent experiments. (E) (left panel) HGS reduced HBV replication in the hydrodynamically injected liver by Southern blot analysis. Each lane represents a liver sample from each mouse. (right panel) HGS reduced HBV DNA in the mouse sera by Southern blot analysis. Data are representative of two independent experiments. (F) HGS reduced the formation of HBV virions and HBsAg particles from pooled mouse sera by 1% native agarose gel electrophoresis at 3 dpi. (G) Liver injurieswere similar between the control and HGS-expressing mice at 1 dpi (p = 0.19 by the Student’s *t*-test), as measured by the serum alanine aminotransferase (ALT) levels.

Next, we attempted to extend the cell culture studies to an *in vivo* experimental setting. Briefly, we hydrodynamically delivered plasmid DNAs of an HBV replicon and an HGS expression vector at various ratios into BALB/c mice by tail vein co-injection. Five mice from each experimental group were sacrificed at 1 or 3 days post-injection (dpi) to monitor the viral protein expression. Exogenous Myc-HGS was readily detected at 1 dpi in mouse liver by Western blot analysis (Fig 4A). Immunohistochemistry (IHC) staining showed that co-transfection with HGS efficiently suppressed HBV core protein expression in the sectioned liver of this mouse model (Fig 4B). Furthermore, co-expression of HGS and HBc proteins in the same hepatocytes became more apparent with an increased dose of HBV replicon (S4 Fig). Consistent with the IHC data, HBsAg and HBeAg in mouse sera were both reduced at 1 and 3 dpi (Fig 4C and 4D). In addition, we collected the intracellular core particle-associated HBV DNA in the liver tissue as well as the extracellular HBV DNA from pooled mouse sera at 3 dpi. The Southern blot results between the liver and serum compartments were consistent with each other (Fig 4E). Again, HBV replication *in vivo* was severely impaired upon HGS overexpression. Similarly, the amounts of HBV virions and HBsAg particles in the sera were significantly reduced upon co-injection with HGS (Fig 4F). We also measured the level of serum alanine aminotransferase (ALT) as a parameter of hepatocellular injury. We detected no significant difference (p = 0.19) in serum ALT levels between the two groups of mice with or without HGS (Fig 4G). Therefore, overexpressed HGS can inhibit viral DNA replication and protein expression in both cell culture and animal model.

### Overexpressed HGS stimulated the release of HBV naked capsid particles

**Fig 5:**
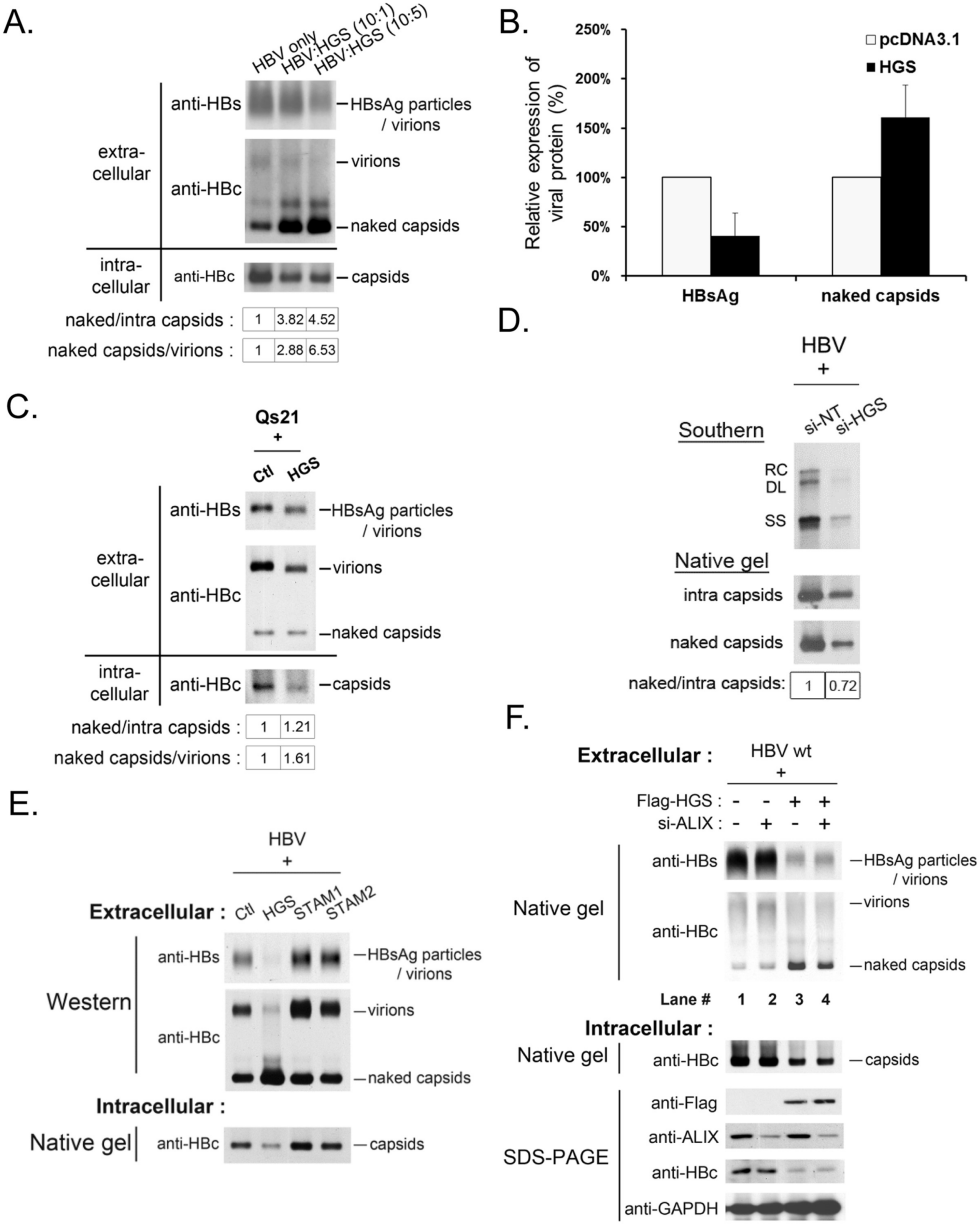
Overexpressed HGS stimulated the release of HBV naked capsid particles. (A) HGS stimulated the secretion of naked capsids in a dose-dependent manner, but not virions or HBsAg particles by native agarose gel electrophoresis. HBV replicon pCHT-9/3091 and HGS expression vector DNA were mixed at different ratios before co-transfection into HuH-7 cells. Secreted HBV particles in supernatants were collected by ultracentrifugation at day 3 post-transfection. The ratios of extracellular capsids over intracellular capsids or virions were quantified by Image J analysis and normalized to the control group. (B) Co-transfection of an HBV replicon plasmid pCHT-9/3091 and an HGS expression vector suppressed HBsAg expression, but strongly stimulated the secretion of naked capsids by ELISA assay (see text for details). Data are representative of two independent experiments. (C) Overexpression of HGS (0.3 μg) in a stable HBV-producing cell line Qs21 significantly reduced virion secretion without an apparent effect on the secretion of naked capsids. The ratio of naked capsids/virions (1.61) suggests that HGS could help maintain the level of naked capsids despite the concurrent reduction in virions. (D) Knockdown of endogenous HGS in HepG2 cells suppressed the secretion of naked capsids, as well as reduced the synthesis of intracellular viral DNA and capsid particles. The ratio of naked/intra capsid particles was quantified by Image J analysis and normalized to the si-NT control group. Data are representative of two independent experiments. (E) Unlike HGS, overexpression of the other two ESCRT-0 factors STAM1 and STAM2, stimulated HBsAg expression and virion formation without an apparent enhancement of the secretion of naked capsids. (F) ALIX, an ESCRT accessory factor, could contribute to the HGS-facilitated secretion of naked capsids. Co-transfection of HGS expression vector (0.3 μg) and si-ALIX (50 nM) in HuH-7 cells reduced the level of secreted naked capsids at day 5 post-transfection in two independent transfection experiments (compare lanes 3 and 4).

Most surprisingly, we observed substantially increased amounts of naked capsids in the supernatant upon HGS overexpression, which is in sharp contrast to the reduced intracellular capsids in cell lysates (Fig 5A, lower panel). Since both HBsAg particles and virions were reproducibly reduced in the presence of a higher dose of HGS (10:5 ratio in Fig 5A), it strongly suggests that the secretion pathways of virions and HBsAg particles are different from that of naked capsids. Both HBeAg and naked capsids can cross-react with antibodies in some commercial ELISA kits of HBeAg. Plasmid pCHT-9/3091 is an HBV replicon without HBeAg production [35–37]. Therefore, one can detect the secreted naked capsids in the medium of pCHT-9/3091 transfected culture without the complication from HBeAg by ELISA. As anticipated, co-transfection of plasmid pCHT-9/3091 with an HGS expression vector suppressed the level of secreted HBsAg (Fig 5B). In contrast, the ELISA signal of naked capsids was increased upon HGS co-transfection. To exclude the possibility of naked capsids released from cell lysis [38], we measured the apoptotic markers, such as caspase 3 activation and PARP1 cleavage, in HGS-overexpressing HuH-7 cells (S5A Fig). We detected neither apoptosis nor cytotoxicity by LDH assay (S5B Fig).

The dual effects of HGS on HBV transcriptional suppression and enhanced naked capsid secretion were further supported in an HBV-producing stable cell line Qs21 (Fig 5C) [39,40], albeit the promotion effect of HGS on naked capsid secretion was less potent in Qs21 cells than that in HuH-7 cells. Upon HGS overexpression, despite the levels of intracellular capsids and secreted virions of Qs21 were significantly reduced, the level of secreted naked capsids remained unchanged relative to the control (Fig 5C). Instead of using an overexpression approach, when si-HGS was co-transfected with HBV replicon into HepG2 cells, we observed a more pronounced reduction in naked capsid secretion relative to the level of intracellular capsids (Fig 5D). Taken together, despite the fact that the HGS effect may slightly vary in different hepatoma cell lines, HGS is required for the secretion of naked capsids.

This HGS effect on the secretion of naked capsids raised an issue of whether the other two known ESCRT-0 components, STAM1 and STAM2, could also behave similarly. To address this issue, we compared side by side the respective effects of HGS, STAM1 and STAM2 on the secretions of viral and subviral particles by the native agarose gel analysis. In contrast to the transcriptional suppression effect of HGS, overexpression of STAM1 and STAM2 resulted in an increased intracellular level of capsids as well as increased secretion of HBsAg particles and virions (Fig 5E). Unlike HGS, we observed no apparent promotion effect of STAM1 and STAM2 on the secretion of naked capsids. Therefore, it appears that HGS is the only component of the ESCRT-0 complex capable of promoting the secretion of naked capsids. Similarly, an ESCRT accessory factor, ALIX, was reported to be involved in the secretion of HBV naked capsids [31]. We therefore tested whether the effect of HGS on the secretion of naked capsids is related to ALIX. As shown in the experiment described in Fig 5F, upon HGS overexpression, the amount of secreted naked capsids was reduced by the si-ALIX treatment (compare lanes 3 and 4, Fig 5F). Therefore, it is possible that ALIX could somehow facilitate the process of HGS-promoted secretion of naked capsids.

### HGS promoted the secretion of naked capsids irrespective of their genome content or maturation status

**Fig 6:**
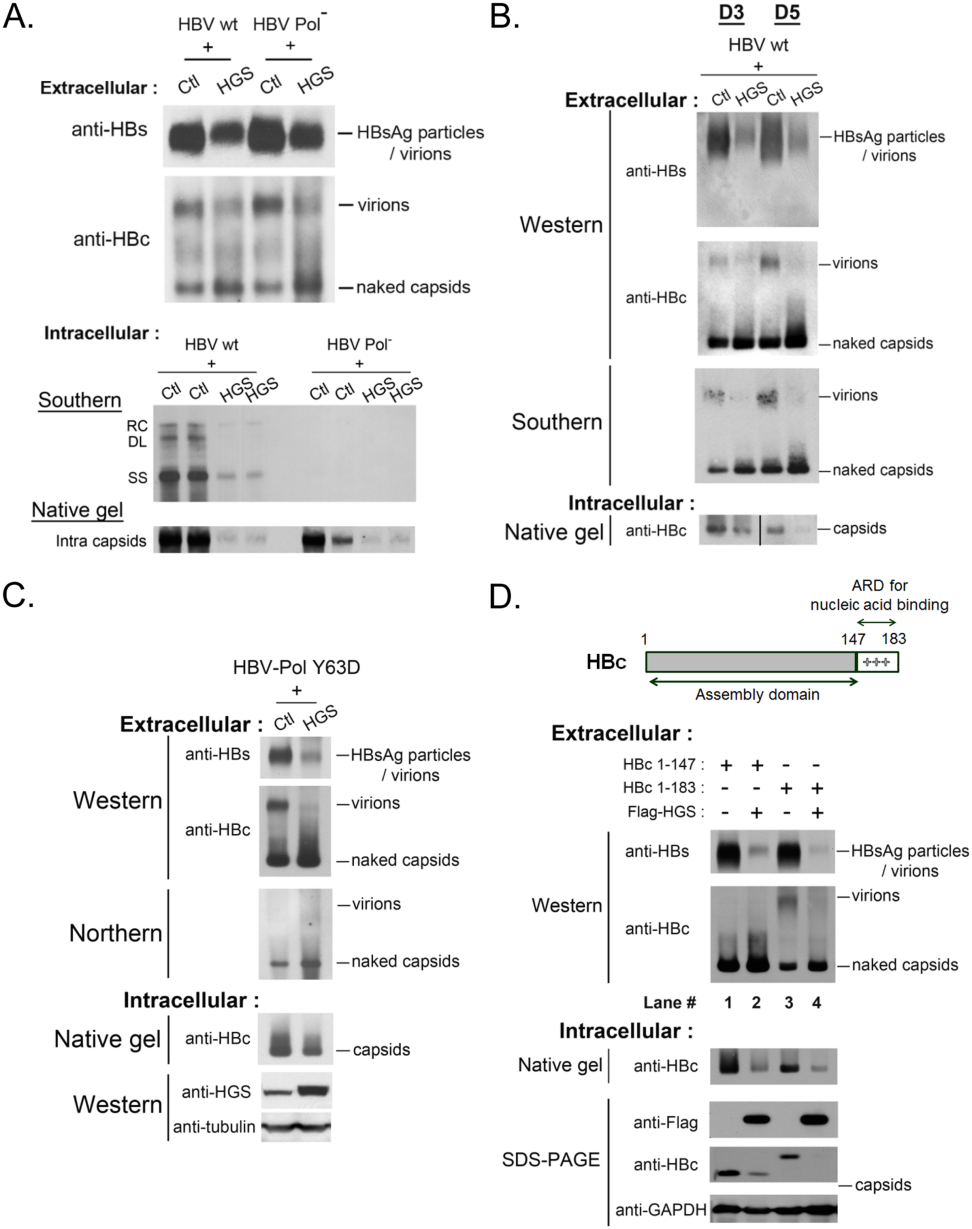
HGS stimulated the secretion of naked capsids regardless of the genome maturation status. (A) (upper panel) HGS can promote the secretion of empty naked capsids in a viral polymerase-independent manner. The polymerase-null missense mutant 2310 is defective in AUG initiation of HBV polymerase. While this mutant is normal in the expression of HBsAg and HBc, it is unable to encapsidate pregenomic RNA due to lack of the polymerase. As shown in the lower panel, this polymerase-null mutant could not replicate itself. (One of the duplicated control samples in native gel was partially lost) (B) HGS co-transfection with wild type HBV into HuH-7 cells increased the secretion of DNA-containing naked capsids of at day 3 and 5 post-transfection. In contrast, HBsAg particles, total virions, as well as virion-associated viral DNA, were all reduced by HGS. Data are representative of two independent experiments. (C) HGS can stimulate the secretion of naked capsids containing viral RNA of HBV polymerase mutant Y63D by Northern blot analysis, despite its suppressive effects on HBsAg particles, total virions, and intracellular capsids. Data are representative of two independent experiments. (D) The arginine-rich domain (ARD) of HBc 147–183 is dispensable for the HGS-enhanced secretion of naked capsids (compare lanes 1–4). The result here also suggests that ARD could play a role essential to the secretion of empty virions (compare lanes 1 and 3).

To better understand the mechanism behind the HGS-promoted secretion of naked capsids in Fig 5, we used various HBV mutants to examine the genome content and maturation status of extracellular naked capsids. The polymerase-null mutant is an HBV dimer containing a mutation at the polymerase intiation codon (nt 2310) [41]. This mutant failed to encapsidate HBV RNA and thus no reverse-transcribed DNA synthesis (Fig 6A, lower panel). Since the increased levels of naked capsids were comparable between wild type HBV and the replication-defective polymerase-null mutant (Fig 6A, upper panel), the stimulatory effect of HGS on naked capsid secretion is independent from HBV polymerase or viral replication. The result here suggested that empty capsids without encapsidated RNA or DNA genome can still be stimulated by HGS for secretion. Next, we demonstrated that DNA-containing wild type naked capsids can also be stimulated by HGS for secretion by Southern blot analysis (Fig 6B). In contrast, the virion-associated viral DNA was significantly reduced in the same HGS co-transfection experiment (Fig 6B). Next, we checked further whether secretion of RNA-containing naked capsids can also be promoted by HGS. A polymerase mutant Y63D [42], which enables RNA encapsidation but fails to synthesize negative-strand HBV DNA, was enrolled in the study. As anticipated, RNA signal was not detectable in the virions of mutant Y63D (Fig 6C), a result consistent with the previous report [26]. Moreover, secretion of RNA-containing naked capsids can be stimulated by HGS (Fig 6C).

Full-length HBV core protein (aa 1–183) consists of two distinct domains (Fig 6D, upper panel). The N-terminal domain of HBc (aa 1–147) alone is necessary and sufficient for capsid assembly [43]. In contrast, the C-terminal domain of HBc (aa 147–183) is an arginine-rich domain (ARD) important for multiple activities, including RNA encapsidation, DNA synthesis, HBc nuclear import and export, as well as charge balance in the capsid interior [43–46]. The results in Fig 6A–6C strongly suggest that HGS can promote the secretion of naked capsids, regardless of their genomic content. This conclusion is reinforced by the experiment using HBc 1–147 in Fig 6D. Like the full-length HBc 1–183, ARD-truncated capsids of HBc 1–147 were by themselves secretable, and could be further promoted for secretion by HGS (compare lanes 1 and 2). Most surprisingly, ARD-truncated HBc 1–147 failed to form empty virions (compare lanes 1 and 3), which were clearly detectable even in the absence of viral replication (Fig 6A and 6C). Herein, we demonstrated a new function of the HBc ARD as a crucial determinant for the formation of empty virions, and most likely, DNA-containing virions as well.

In our preliminary attempt to quantitatively compare the effects of HGS on the secretion of various viral and subviral particles, we measured the signal intensities of these particles on the native gels (S6 Fig). Overall, the enhanced secretion of HBV naked capsids by HGS appears to be associated with the concurrent reduction in virion secretion (Fig 6 and S6 Fig).

### Interaction between HGS and HBV core protein is ubiquitin-independent

**Fig 7:**
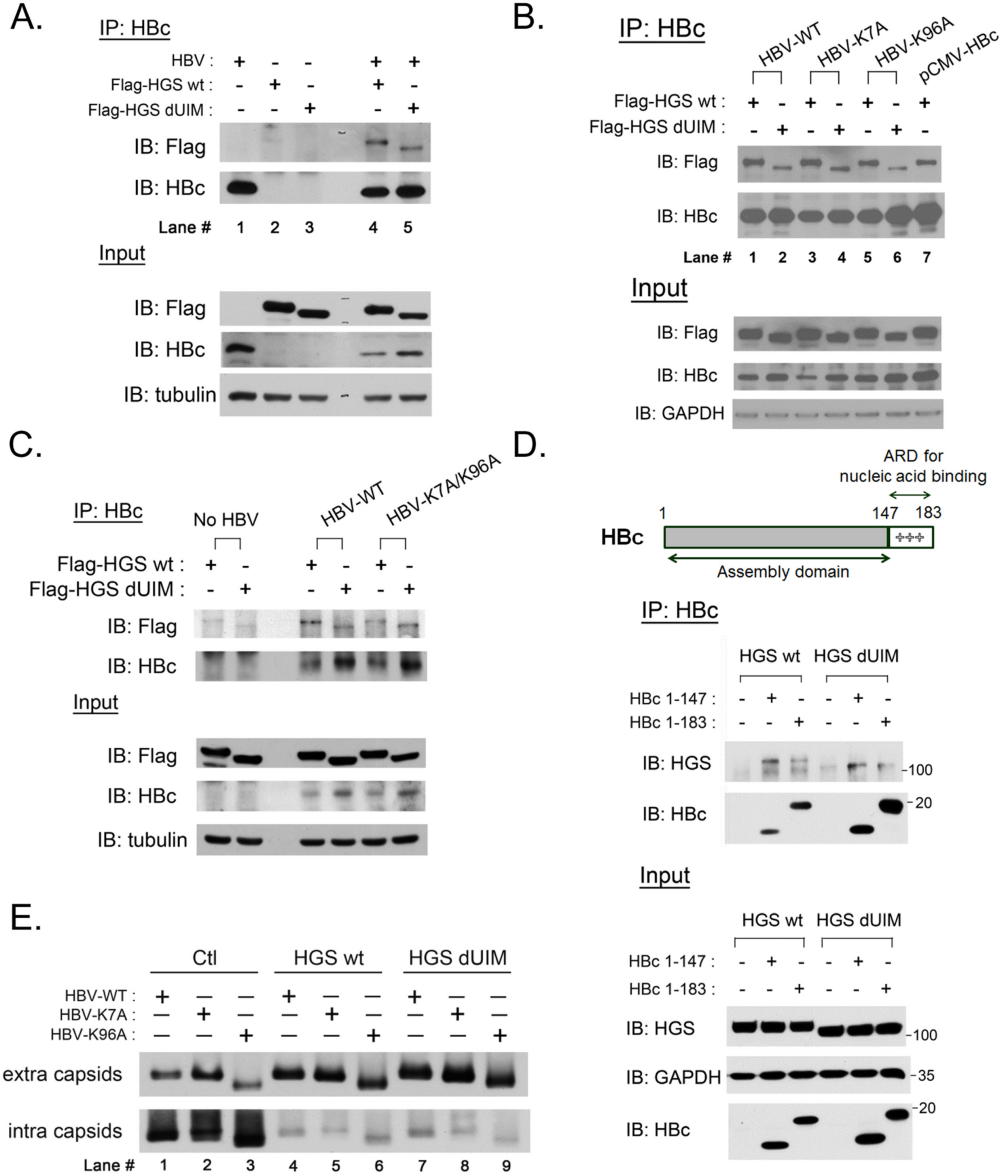
ESCRT-0 protein HGS associated with HBV core protein through an ubiquitin-independent recognition. (A) The association between HGS and HBc proteins was identified by the co-IP assay at day 3 post-transfection. The HBV replicon plasmid pCHT-9/3091 and an HGS expression vector (2:1 ratio) were co-transfected into HuH-7 cells. Deletion of the known ubiquitin interaction motif (UIM, aa 257–277) of HGS exhibited no effect on its association with HBc. (B) There are only two lysine residues K7 and K96 in HBc which could serve as candidate ubiquitination sites. Lysine to alanine substitutions at these two sites (K7A and K96A) did not abrogate the association between HBc and HGS, with or without UIM. (C) A lysine-free HBc double mutant K7A/K96A maintained the associations with HGS and HGS dUIM in the co-IP assay, suggesting that the association between HBc and HGS is ubiquitination-independent. (D) ARD-truncated mutant HBc 1–147 containing the N-terminal capsid assembly domain was necessary and sufficient for its association with wild type HGS and HGS dUIM mutant. (E) HGS can efficiently promote the secretion of naked capsids in an ubiquitin-independent manner in HuH-7 cells at day 5 post-transfection. No difference in the enhanced secretion of naked capsids was detected between wild type and mutant HBV, or between wild type and mutant HGS, despite the fact that intracellular capsids were all significantly diminished upon wild type and mutant HGS overexpression.

HGS is known to serve as the initiator for the ESCRT cascade, mainly through recognizing ubiquitinated substrates for endosomal trafficking. To test whether HBV core protein could be recognized by HGS, we examined the physical association between HBc and HGS by a co-immunoprecipitation (co-IP) experiment. As shown in Fig 7A, HBV core protein can indeed be associated with the exogenous tagged-HGS. Since the ubiquitin-interacting motif (UIM) of HGS was supposed to recognize the cargo [47], we deleted the UIM region (aa 257–277) from HGS, and performed the co-IP experiment with the wild type HBV core protein. Overexpression of UIM-truncated HGS (HGS dUIM) remained inhibitory to HBV replication as detected by Southern blot (S7A Fig), albeit the potency of inhibition was somewhat reduced relative to the wild type HGS. We noted that the steady state level of HGS dUIM was lower than that of wild type HGS, and thus might in part contribute to the reduced potency in suppressing HBV transcription (S7B Fig). Consequently, overexpression of HGS dUIM resulted in a higher level of HBc relative to the wild type HGS. As shown in Fig 7A, the co-IP experiment demonstrated that the associations between HBc and HGS, with or without UIM, were quite similar (compare lanes 4 and 5).

In a reciprocal experiment, we asked whether mutant HBc, containing lysine-to-alanine substitutions at amino acids 7 or/and 96, can still be associated with the wild type HGS protein. By comparing the HGS intensities in lanes 1, 3, and 5 in Fig 7B, we observed no significant difference in the associations of wild type HGS with wild type HBc, mutant HBc K7A, or mutant HBc K96A, respectively. Similarly, we detected no appreciable difference in mutant HGS intensities between lanes 2, 4, and 6 of Fig 7B. Again, when the HBc K7A/K96A double mutant was used in this co-IP experiment, we obtained a similar result (Fig 7C). Taken together, it suggests that the only two lysine residues at amino acids 7 and 96 of HBc, are not important for the association between HBc and HGS. Thus, the HBc protein appeared to associate with the HGS protein in an ubiquitin-independent manner.

Having demonstrated that the secretion of naked capsids of ARD-truncated HBc 1–147 could be stimulated by HGS (Fig 6D), we further demonstrated here that HBc 1–147 can co-immunoprecipitate the HGS protein, with or without UIM, as efficiently as the wild type HBV with full-length HBc 1–183 (Fig 7D). Taken together, the capsid assembly domain of HBc alone is necessary and sufficient for its association with HGS.

As shown in Fig 6, we demonstrated that HGS can promote the release of naked core particles, irrespective of their genome contents. Here, we demonstrated that the secretion of naked HBV capsids can be promoted by HGS in a manner independent of the UIM domain of HGS as well as the lysine residues of HBc (Fig 7E). Interestingly, we noted that both intracellular and extracellular capsids of HBc K96A migrated faster than those of wild type HBc and mutant HBc K7A (Fig 7E). This result indicates that the alteration of the charge or conformation at amino acid 96 of HBc can affect the mobility of capsids on the native gel. In summary, the UIM domain of HGS does not seem to play an essential role in the physical and functional relationships between HBc and HGS.

### Cytoplasmic HBc co-localized with HGS predominantly near the cell periphery, but not the enlarged endosomes with a punctate structure

**Fig 8:**
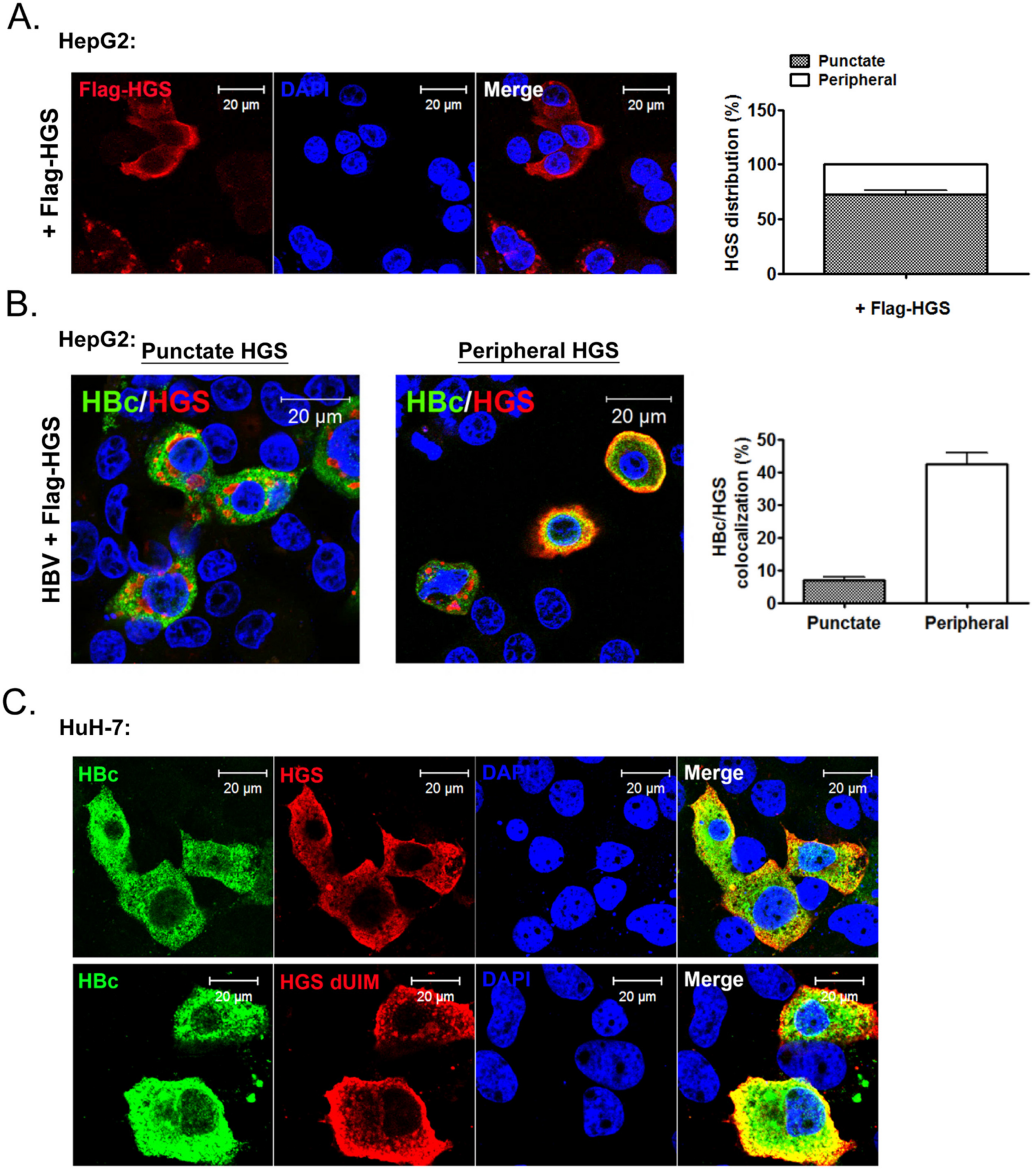
HBc co-localized with HGS mostly near the cell periphery, but not within the punctate structure of HGS. (A) Confocal microscopic analysis of the Flag-HGS protein alone revealed two distinct patterns of subcellular localization in HepG2 cells at day 2 post-transfection. Approximately 70% of the HGS-transfected cells (anti-Flag) exhibited a punctate structure in the cytoplasm (right panel). The remaining 30% of cells exhibited a diffuse HGS distribution in the cytoplasm with a more enriched concentration toward the cell periphery. (B) Confocal microscopic analysis of the co-localization pattern between HBc (anti-HBc, Dako) and Flag-HGS (anti-Flag). An HBV replicon plasmid pCHT-9/3091 and an HGS expression vector were co-transfected into HepG2 cells. Co-localization was observed mainly near the cell periphery, but less often with the punctate structure. Quantification of the relative abundance of the co-localization patterns (periphery vs. puncta) was shown in the right panel. Data above are representative of three independent experiments. (C) A similar HBc and HGS protein co-localization pattern was observed between wild type HGS and mutant HGS dUIM in HuH-7 cells.

Overexpression of HGS is known to perturb the post-endocytic sorting events by inducing the formation of enlarged endosomes and preventing lysosomal trafficking [48,49]. To visualize where HGS and HBc could encounter with each other at the subcellular level, we examined the localizations of HBc alone, HGS alone, as well as HBc/HGS, in HepG2 cells by confocal immunofluorescence imaging. Upon HBV transfection alone, HBc showed a pan-cytoplasmic staining pattern with rare nuclear accumulation (S8A Fig). The endogenous HGS, known to be targeting the PI3P-enriched endosome membrane [50], showed only sporadic co-localization with HBc (S8A Fig). This negative result is in part due to the low level of endogenous HGS protein in both HepG2 and HuH-7 cells. When exogenous HGS was expressed in HepG2 cells, we observed two different localization patterns (Fig 8A). A large proportion (~70%) of the Flag-HGS-expressing cells exhibited HGS accumulation in the punctate structure, which is partially co-localized with an early endosome marker EEA1 as described previously (S8B Fig) [50]. However, a minor proportion (~30%) of the Flag-HGS-expressing cells showed a more enriched distribution of HGS toward the cell periphery in the cytoplasm (Fig 8A). Similar distributions of Myc-HGS around both cell periphery and punctates were observed in sectioned liver upon the hydrodynamic delivery (S9 Fig). Interestingly, we noted that the majority of the HGS-positive puncta were not strongly associated with HBc in HepG2 cells co-expressing HBV and Flag-HGS (Fig 8B). In contrast, those endosome-free, peripheral HGS were predominantly co-localized with HBc and exhibited a halo-like distribution around the cell periphery (Fig 8B). This HBc/HGS co-localization pattern was also observed in HuH-7 cells (Fig 8C, upper panel). Like the wild type HGS, UIM-domain truncated HGS displayed a pattern more peripheral-enriched than punctate-associated (Fig 8C, lower panel). Again, the UIM domain of HGS does not play a role in the naked capsid secretion as well as in the association and co-localization with HBc.

## Discussion

### ESCRT cascades in HBV transcription and replication

We identified a number of ESCRT factors (18 out of 30) among different ESCRT complexes, which can affect HBV replication and gene expression (Fig 1 and Table 1). This phenomenon suggests that the ESCRT cascades somehow could be involved in HBV transcription and replication. One of the possible explanations for the lack of effect on HBV from the other factors (12 out of 30), could be related to the presence of functionally redundant isoforms of ESCRT factors, e.g., STAM1 and STAM2 in ESCRT-0 [33], CHMP4A, 4B, and 4C in ESCRT-III [15]. Since all of the ESCRT-0 factors were required for HBV replication, it suggests that the ESCRT-0 complex *per se* played an important role in HBV propagation. Under- or over-expression of the key component HGS in ESCRT-0 resulted in the suppression of HBV RNA transcription, DNA replication, as well as virion production (Figs 2 and 3). Furthermore, altered expression of HGS could also inhibit HBV replication in an animal model (Fig 4). An optimum level of HGS is required for HBV transcription in the mammalian system. While CMV and native HBV promoters are equally sensitive to the inhibitory effect of overexpressed HGS (Fig 3 and S3 Fig), we noted a reproducible difference in their relative sensitivity to the effect of si-HGS (Fig 2E and S2 Fig). Since HGS is not a nuclear protein, the exact mechanism of its function in regulating transcription still needs to be investigated in the future. Overall, the relationship between HGS and HBV is strikingly similar to what we had observed previously in the case of VPS4, i.e., both too much and too little wild type VPS4, could equally dampen viral replication.

In addition to HGS, silencing of other ESCRT factors, such as STAM1, UBAP1 and CHMP2A, could severely blunt HBV transcription in HepG2 (Fig 1B). It remains to be investigated whether these factors could exert some kind of non-canonical ESCRT functions in HBV-replicating hepatocytes. Several ESCRT factors are known to be versatile, multifunctional, with pleiotropic effects on multiple targets. For example, the implications of ESCRT complexes in signaling pathways were well-documented [51–53]. In the case of the ESCRT-0 component, it could serve as a positive and negative regulator of RTK signaling during Drosophila development [54]. Overdosed HGS was also reported to attenuate BMP-dependent transcriptional responses [55]. Similarly, some ESCRT members were found to play a role in the yeast transcriptional control [56]. In addition, an ESCRT-II component has been implicated in RNA trafficking through an RNA-binding protein Staufen in *Drosophila melanogaster* and HIV [32,57]. Finally, it is worth mentioning that Archaea and plant viruses were also reported to involve the ESCRT machinery for viral replication recently [58–60].

Depletion of ESCRT factors showed some autophagy-related phenotypes with the accumulation of abnormal endosomes and autophagosomes [61]. This autophagic pathway was shown to be required for HBV DNA replication both *in vitro* and *in vivo* [62,63]. It remains to be investigated whether there is any potential effect of autophagy on the ESCRT cascade, or vice versa, in hepatocytes. Nevertheless, it is conceivable that autophagy and ESCRT machineries could have close interplay in their membrane dynamics, which somehow plays an important role in HBV propagation.

### Roles of the ESCRT complexes in the secretion of HBV naked capsids

We discovered here that the egress of HBV naked capsids can be promoted by HGS, irrespective of their genomic or empty contents. This phenomenon appears to be HGS-specific, rather than ESCRT-0 specific, since we detected no similar effect on naked capsid secretion by STAM1 and STAM2 (Fig 5E). Another ESCRT-related factor ALIX was shown to influence the secretion of naked capsids in an ESCRT-independent manner [31]. While we detected no similar reduction in the secretion of naked capsids by si-ALIX treatment in our experimental setting, we demonstrated here that ALIX can indeed facilitate the process of HGS-promoted secretion of naked capsids (Fig 5F). ALIX is known to interact with an ESCRT-I factor TSG101 through its proline-rich domain [64,65]. Similarly, HGS and TSG101 can make multiple independent contacts with each other [66]. Thus, it is tempting to speculate that ALIX and HGS could somehow work together on the regulation of naked capsid secretion.

### An ubiquitination-independent recognition between HBc and HGS

While HGS is better known for its ability to target the ubiquitin-modified cargoes [67,68], it has also been shown to recognize a motif of clustering hydrophobic amino acids in cytokine receptors IL-2Rb and IL-4Ra, in an ubiquitin-independent manner [69,70]. Similarly, our result from the co-IP experiment suggested that the HGS protein could be associated with the assembly domain of HBc in a manner independent of the potential ubiquitination sites in both HGS (UIM domain) and HBc (K7, K96) (Fig 7). In addition, the UIM domain of HGS does not play a role in the naked capsid secretion as well as in the co-localization with HBc (Figs 7 and 8).

### Secretion routes of HBV naked capsids

Interestingly, in both HepG2 and HuH-7 cells, we found that the cytoplasmic HBc was predominantly co-localized with HGS near the cell periphery. This result raised an issue of whether HGS could promote the event of either capsid assembly or capsid secretion *per se*. It is generally believed that HBV capsid assembly occurs early after translation of core and polymerase mediated by the ribosomal machinery, and is not known to occur at a site near the plasma membrane later in HBV life cycle. Consistent with this conceptual context, we detected no increased intracellular capsid particles by HGS using the native agarose gel assay (relative to the intracellular amount of HBc protein monomer on SDS-PAGE). In brief, we are in favor of the idea that the co-localization between HGS and HBc near the cell periphery could be related to the promotion of secretion, rather than assembly, of naked capsids. Indeed, HGS can be recruited to the plasma membrane by endocytic FEI (FCHO-1, EHS-1, and ITSN-1) adaptor complexes [71]. Furthermore, HGS was known to be important for the release of exosomes in dendritic cells and HuH-7 cells [72,73]. It remains to be tested whether inhibitors for exosome production could reduce the HGS-mediated egress of naked capsids. Recently, another class of extracellular vesicles, known as ectosomes, were assembled and released directly from the plasma membrane, instead of through the exocytosis route from MVBs [74]. Notably, HGS was also required for efficient ectosome production [75]. Taken together, it remains to be addressed whether the egress of naked capsids could be related to the release of ectosome vesicles.

### Distinct secretion routes of HBV virions vs. naked capsids

The secretion of naked capsids has remained a mystery since naked capsids can hardly be detected in HBV-infected patients, albeit they can be commonly detected in the cell culture of various hepadnaviruses [25,35,76–78]. One possible explanation for the lack of detection of naked capsids *in vivo*, is the very rapid clearance of highly immunogenic naked capsids by the potent and long-lasting anti-core antibody in the blood circulation of most HBV-infected patients [79,80]. Similarly, in the hydrodynamic mouse model, anti-core antibody emerged very early around 3 dpi [81]. Our results showed that HGS can specifically stimulate the release of only naked capsids, but not HBsAg particles or virions (Figs 5 and 6). In fact, both virions and HBsAg particles are known to be assembled and exported via the ER-Golgi route.

Wild type hepadnaviruses preferentially secrete virions containing mature genome (double-stranded DNA) over immature genomes (RNA or SS DNA) [82]. One exception to this dogma is the frequent naturally occurring HBc variant I97L which can secrete virions containing SS DNA as efficiently as those containing DS DNA [76,83]. On the contrary, secreted HBV naked capsids at the higher density fractions contain predominantly SS DNA [76]. It is noteworthy that naked capsids can be stimulated to egress by HGS in a manner independent from its genomic content (Fig 6). Consistent with this finding, we demonstrated that empty naked capsids of ARD-truncated HBc 1–147 can be promoted for secretion by HGS. Unexpectedly, we discovered that HBc ARD was crucial for empty virion secretion, probably by serving as a signal to the envelopment machinery. It is therefore tempting to speculate here that HBc ARD could serve as or contribute to the long sought-after “genome maturation signal” important for hepadnavirus capsid envelopment and virion egress [82]. Taken together, the lack of stringency or selectivity in the secretion of naked capsids again lends support for the possibility that the egress routes between virions and naked capsids are distinct from each other.

### HGS and HIV-1

In our study here, overexpression of HGS can reduce HBV virion release, in part by promoting the secretion of naked capsids. It is known that during HIV-1 morphogenesis, Gag protein can mimic HGS in recruiting TSG101 of the ESCRT-I complex, and thus bypass ESCRT-0 for virus budding and virion release [84]. Thus, overexpression of wild type HGS can potently interfere with HIV-1 production, albeit no discernible effect of HGS on the production of Moloney murine leukemia virus was observed [66]. It should also be mentioned here that HGS could affect HIV morphogenesis in another way. As an important arm of innate immunity against viral infection, BST-2/tetherin is a host factor capable of restricting the release of virions. With the help of HGS, this innate immunity restriction can be efficiently counteracted by an HIV-1 encoded protein vpu [85]. Recent studies reported the importance of BST-2/tetherin in restricting HBV virion secretion, but not the secretion of naked capsids [86,87]. Unlike tetherin, HGS does play a positive role for the secretion of HBV naked capsids.

Collectively, our work demonstrated two major effects of HGS on HBV propagation. One is by dampening HBV enhancer-promoter transcriptional activity. The other is by promoting the secretion of empty and nucleic acids-containing capsids, leading to a diminished pool of intracellular capsids with ongoing viral DNA synthesis. It remains an appealing idea to investigate in the future whether one can treat viral hepatitis B by promoting excessive and non-selective egress of intracellular capsids.

## Materials and Methods

### Ethics statement

All animal experiments were conducted under protocols approved by Academia Sinica Institutional Animal Care & Utilization Committee (ASIACUC permit number 12-02-322). Research was conducted in compliance with the principles stated in the Guide for the Care and Use of Laboratory Animals, National Research Council, 1996.

### Cell lines and reagents

Human hepatoma cell lines HepG2 (from Dr. B. Knowles at Wistar Institute, Philadelphia) [88] and HuH-7 (from Dr. M. Lai at Academia Sinica, Taiwan) [89] and rat hepatoma cell line Qs21 [39,40] were grown in Dulbecco’s modified Eagle medium (DMEM) supplemented with 100 U of penicillin per ml, 100 μg of streptomycin per ml, and 10% fetal bovine serum (complete DMEM medium). SMARTpool siRNAs specifically targeting human ESCRTs component genes as well as non-targeting siRNA control (D-001810) were all purchased from Dharmacon Inc, USA. Lipofectamine 2000 (Invitrogen) used for siRNA and plasmid DNA co-transfection, and PolyJet reagent (SignaGen Laboratories) used for DNA plasmid only transfection, were both performed according to the manufacturers' instructions. MTT viability assay (Promega), LDH Cytotoxicity Assay (Promega) and the HBsAg/HBeAg ELISA kit (General Biologicals Cooperation, Taiwan) were performed in accordance to the vendor's protocols.

### Plasmids

The tandem dimer construct of wild type HBV (ayw) and the polymerase-defective mutant construct 2310 were as described elsewhere [41]. The wild type HBV (adr) plasmid [83] was used in our animal study via hydrodynamic delivery. Plasmid pCHT-9/3091 is an HBV (ayw) 1.2 mer construct without the expression of precore and HBeAg [35]. HBV core mutants at lysine 7 (K7A), lysine 96 (K96A), double mutant at lysine7 and 96 (K7A/K96A) as well as the polymerase mutant at tyrosine 63 (Y63D) residues were generated from the parental plasmid pCHT-9/3091 by QuickChange II XL Site-Directed Mutagenesis kit (Stratagene). Another C-terminal deleted core mutant, HBc 1–147, was engineered by introducing a stop codon at amino acid 148 in the pCHT-9/3091 template. The Myc-Flag-tagged HGS, STAM1, and STAM2 expression vectors used in the study were all purchased from OriGene. Plasmid HGS dUIM was derived from the wild type HGS backbone by deleting a putative ubiquitin-interaction motif at amino acid 257–277 by PCR amplification. All of the mutant plasmids above were confirmed by DNA sequencing. The reporter plasmid pGL3-EnhII-Cp, driven by HBV enhancer II and core promoter region (ayw, nt 1600–1864), and the control pRL-TK plasmid (Promega), were kindly provided by Dr. Fang Liao at Academia Sinica, Taiwan.

### RNA extraction and real-time qPCR

RNA samples were prepared with TRIzol Reagent (Invitrogen) and reverse transcribed into cDNA using a High Capacity cDNA Reverse Transcription Kit (Applied Biosystems). All the gene-specific primers were designed according to the online NCBI Primer-BLAST software. To detect the virion-associated HBV DNA, culture supernatant was first immunoprecipitated by anti-HBs (Dako) antibody, followed by DNA extraction with QIAamp MinElute Virus Spin Kit (QIAGEN). Expression of cellular mRNA or viral DNA was quantified by SYBR green and ABI 7500 Real-Time PCR System (Applied Biosystems). The PCR primers used for detection of HBV DNA in virions are HBV-322F: 5’ CCAACCTCCAATCACTCACC 3’ and HBV-440R: 5’ AACAAGAAGATGAGGCATAGC 3’.

### Southern and northern blot analysis

In general, an HBV replicon plasmid and respective pooled siRNAs (50 nM) were co-transfected into HepG2 or HuH-7 cells with Lipofectamine 2000 reagent in duplicates. Cells were harvested in parallel at day 3 or 5 post-transfection. Core-associated DNA extraction, Southern and Northern blot procedures were performed as described elsewhere [76]. The digoxygenin-labeled HBV specific probe (nt 1521–3164) was used for HBV DNA/RNA detection. Signal of HBV replication intermediates detected in Southern blot was quantified by Image J software (National Institutes of Health).

### Antibodies

Anti-HBc antibodies of rabbit polyclonal (Dako) and mouse monoclonal origins (Hyb-3120, Institute of Immunology), were used for immunoblotting and immunofluorescence assays. A rabbit anti-HBc antibody was used in co-IP assay [45]. Other antibodies for HGS, STAM1, GAPDH, α-tubulin, ALIX, caspase 3, PARP1, EEA1 as well as peroxidase-labeled secondary antibodies were all obtained from ICON-GeneTex, Taiwan. Anti-Myc (9E10) and Rhodamine/FITC-labeled secondary antibodies were from Santa Cruz Biotechnology. Anti-FLAG M2 antibody (Sigma-Aldrich), anti-STAM2 (Abcam), and anti-HBs (Dako) were all commercially available.

### HBV particles preparation and native agarose gel electrophoresis

Culture medium containing HBV particles was clarified by centrifugation at 3,000 g for 15 minutes, followed by 20% sucrose cushion in Beckman SW28 rotor at 26,000 rpm for 16 hours. Pelleted HBV particles were resuspended in TNE (20 mM Tris pH 7.5, 150 mM NaCl, 1 mM EDTA) buffer, and further digested by micrococcal nuclease/DNaseI (NEB) to eliminate the residual transfected DNA. Aliquots from intracellular nucleocapsids and extracellular HBV particles were separated by 1% agarose gel prepared in Tris-borate-EDTA (TBE) buffer. Particles within the gels were then transferred by 0.05% SDS-containing 2X SSC buffer to nitrocellulose membrane for protein and RNA detections. For DNA detection, gels were denatured in alkaline buffer for 30 minutes before being transferred to membrane. The digoxygenin-labeled HBV specific probe (nt 1521–3164) was used for nucleic acid hybridization. Western blot analysis for capsid expression was performed as previously reported [76].

### Reporter assay

The reporter assay was performed in experiments with knockdown or overexpression of HGS. In the former case, 50 nM SMARTpool siRNAs, 0.1 μg pGL3-EnhII-Cp, and 0.15 μg pRL-TK control plasmid were mixed together and co-transfected into HepG2 cells in 24-well plate in triplicates. In the latter case, 0.025–0.1 μg Myc-Flag-HGS, 0.1 μg pGL3-EnhII-Cp and 0.15 μg pRL-TK were prepared for co-transfection in HepG2 and HuH-7 cells. After 72 hours post transfection, the firefly luciferase expression driven by the HBV promoter was first normalized to the renilla luciferase expression driven by the TK promoter. The luciferase activities were measured by Dual-Glo Luciferase Assay System (Promega).

### Hydrodynamics-based animal study and samples preparation

BALB/c mice at 6–8 week old were purchased from the National Laboratory Animal Center (Taipei, Taiwan). Mice were hydrodynamically injected with 14 μg HBV DNA with either 6 μg Myc-Flag-HGS or control pcDNA 3.1 (Life Technologies) vectors. Briefly, 20 μg DNA were pre-mixed with normal saline in a volume equivalent to 8–10% body weight and injected via tail vein within 5–7 seconds. Intrahepatic HBcAg and exogenously expressed HGS were analyzed by immunohistochemical staining and Western blot. The serum samples were assayed for HBsAg, HBeAg, and serum alanine aminotransferase (ALT) level at indicated dpi. Aliquots of serum samples were concentrated by Beckman MLS-50 rotor at 35,000 rpm for 4 hours. HBV DNA extracted from liver sections and pooled serum samples at 3 dpi were also subjected to Southern blot analysis.

### Immunohistochemical (IHC) staining

Sectioned liver tissues were fixed by 10% neutral buffered formalin and stained with hematoxylin and eosin (H&E) by the Pathology Core Laboratory, IBMS, Academia Sinica. After standard procedures for deparaffinization and rehydration, the endogenous peroxidase was quenched by 3% H_2_O_2_/PBS. HBV core antigen and HGS were recognized by respective antibodies, followed by incubation of peroxidase-conjugating secondary antibody. The staining results from diaminobenzidine (DAB) system (Dako) reflected the expression level of each antigen. The nuclei of liver sections were also counterstained with Mayer's hematoxylin (J.T. Baker).

### Co-immunoprecipitation (co-IP) assay

Transfected cells were lysed with IP buffer: 20 mM Tris pH 8.0, 120 mM NaCl, 0.2% NP-40, 1 mMEDTA, 50 mM NaF, 1 mM Na3VO4 and protease inhibitor cocktails (Calbiochem). Equal amount of cell lysate for each immunoprecipitation reaction was pre-incubated with anti-HBc antibody for 2 hours at 4°C, and then mixed with protein A/G Dynabead (Invitrogen) by gentle rotation for another 2 hours. Beads-protein complexes were washed 3 times by IP buffer before performing SDS-polyacrylamide gel electrophoresis (PAGE). In addition, 1/30 volume of cell lysates was loaded per lane as the input control.

### Immunofluorescence assay (IFA)

Transfected HepG2 and HuH-7 cells were collected at day 2 post-transfection and fixed in 4% paraformaldehyde in PBS for 30 minutes at room temperature. Subsequent procedures were performed as described previously [45]. The fluorescent images were captured by Zeiss LSM510 confocal microscopy.

### Accession numbers

The accession numbers for genes mentioned in this study are shown here. Wild type HBV serotype ayw (GenBank Accession No. J02203/ V01460), HBV serotype adr (GenBank Accession No. AY123041), HGS (GenBank Accession No. NM_004712), STAM1 (GenBank Accession No. NM_003473), and STAM2 (GenBank Accession No. NM_005834).

## Supporting Information

S1 Fig Cell viability and the siRNA knockdown efficacies of various ESCRT siRNAs in HepG2 cells were determined by MTT assay and real-time PCR. (A) A plasmid of HBV genomic DNA dimer (1 μg) and each pooled siRNAs (50 nM) against various ESCRT factors were co-transfected into HepG2 cells. On day 5 post-transfection, no apparent cytotoxicity upon siRNA treatment was observed by the MTT assay. (B) The RNA expression of ESCRT factors can be successfully suppressed by siRNA treatment. Expression of VPS37C, MVB12A/B and CHMP4C, were not detectable in HepG2 cells. Data shown here are the averages of three independent experiments.
(DOCX)

S2 Fig No effects of si-HGS on the reporter activity driven by a TK promoter and viral replication driven by a CMV promoter. (A) As an internal control for the transfection efficiencies in Fig 2E, a renilla luciferase reporter plasmid driven by a TK-promoter was included in the co-transfection experiment with the firefly luciferase reporter. No effect of si-HGS on the renilla activity was detected. Y-axis values represent the relative renilla luciferase activity in si-HGS-treated samples over the samples treated with non-targeting control siRNA. Data shown here are representative of at least three independent experiments. (B) Relative to the HBV tandem dimer replicon, treatment with si-HGS resulted in no appreciable effect on HBV replication by using a replicon plasmid pCHT-9/3091, whose transcription is driven by a CMV promoter.
(DOCX)

S3 Fig Overexpression of HGS significantly suppressed viral transcription and replication driven by the HBV native promoter. Overexpression of HGS strongly reduced the levels of HBV DNA, RNA, and capsid particles. Plasmid DNAs of an HBV tandem dimer (ayw) and an HGS expression vector were co-transfected at a 2:1 (w/w) ratio into HepG2 and HuH-7 cells. On day 5 post-transfection, viral DNA synthesis, cytoplasmic viral RNA and intracellular capsid particles were examined by Southern, Northern, and native agarose gel electrophoresis. Similar to the CMV promoter (Fig 3A), the native HBV promoter of the tandem dimer plasmid is sensitive to the inhibitory effect of overexpressed HGS.
(DOCX)

S4 Fig Highly efficient co-expression of HBc and Flag-HGS proteins was detected by IHC in hepatocytes of serially sectioned liver in hydrodynamically injected mice. Six to eight week old BALB/c mice were tail-vein injected with 30 μg DNA of an HBV replicon pCHT-9/3091 and 6 μg DNA of an Flag-HGS expression vector. IHC analysis from serially sectioned liver showed that HBc (anti-HBc, Dako) and HGS (anti-Flag) proteins were highly co-expressed in the same hepatocytes at 1 dpi, as indicated by the red circles.
(DOCX)

S5 Fig Neither apoptosis nor cytotoxicity was detected in HGS-overexpressing cells. (A) HuH-7 cells were co-transfected with an HBV replicon pCHT-9/3091 and an HGS expression vector. On day 5 post-transfection, apoptosis was monitored by Western blot analysis of apoptotic markers (activated caspase 3 and PARP1 cleavage). (B) Cytotoxicity was measured by the lactate dehydrogenase (LDH) assay. No apparent apoptosis or cytotoxicity was observed. Data are representative of two independent experiments.
(DOCX)

S6 Fig Quantitative comparisons of the effect of overexpressed HGS on various viral and subviral particles in HGS-transfected HuH-7 cells. The ratios of banding intensities among various viral and subviral particles were quantified by Image J analysis. An estimate for the differential effects of HGS is summarized below: naked capsids: empty virions: HBsAg particles: complete virions = 1.5X increase: 1.6X decrease: 2.5X decrease: 3X decrease. Empty virions were calculated according to the data from polymerase mutants (Y63D and 2310), while complete virions were based on the Southern blot data using native agarose gel assay (Fig 6).
(DOCX)

S7 Fig UIM domain-truncated HGS (HGS dUIM) exhibited a reduced potency in suppressing HBV transcription and viral replication. (A) HGS dUIM maintained the suppression effect on viral replication, albeit its potency appeared to be weaker than that of the wild type HGS. HepG2 cells were co-transfected with HBV dimer and HGS expression vectors at a 2:1 (w/w) ratio. Five days post-transfection, viral DNAs were harvested and compared by Southern blot analysis. (B) Overexpression of wild type or mutant HGS inhibited HBV EnhII-Cp activity. The plasmid of pGL3-EnhII-Cp (as depicted in Fig 2E), control plasmid pRL-TK, and HGS expression vectors, were co-transfected into HepG2 and HuH-7 cells. Two days post-transfection, cell lysates were subjected to Western blot and reporter analysis.
(DOCX)

S8 Fig The localization patterns of endogenous and exogenous HGS were examined in HepG2 cells. (A) An HBV replicon pCHT-9/3091 was transfected into HepG2 cells at 48 hours before IFA. Endogenous HGS showed a punctate distribution of weak signal intensity and was sparsely co-localized with the cytoplasmic HBc (anti-HBc, Hyb-3120). (B) The majority of Flag-HGS were clustering into an enlarged punctate structure in the cytoplasm, which partially co-localized with an endosome marker EEA-1.
(DOCX)

S9 Fig Both punctate (white arrow) and peripheral (black arrow) localizations of the exogenous HGS protein can be observed by immunohistochemistry (IHC) in sectioned livers of BALB/c mice hydrodynamically injected with an HGS-expression vector. (DOCX)
