# Supplementary material for: The Dual Role of an ESCRT-0 Component HGS in HBV Transcription and Naked Capsid Secretion
Source: PLoS Pathog. 2015 Oct 2;11(10):e1005123. doi: 10.1371/journal.ppat.1005123 (PMC4592276; doi:10.1371/journal.ppat.1005123)
Supplement: S1 Fig — (A) A plasmid of HBV genomic DNA dimer (1 μg) and each pooled siRNAs (50 nM) against various ESCRT factors were co-transfected into HepG2 cells. On day 5 post-transfection, no apparent cytotoxicity upon siRNA treatment was observed by the MTT assay. (B) The RNA expression of ESCRT factors can be successfully suppressed by siRNA treatment. Expression of VPS37C, MVB12A/B and CHMP4C, were not detectable in HepG2 cells. Data shown here are the averages of three independent experiments. (DOCX) [file ppat.1005123.s001.docx]

**S1 Fig Cell viability and the siRNA knockdown efficacies of various ESCRT siRNAs in HepG2 cells were determined by MTT assay and real-time PCR**

(A) A plasmid of HBV genomic DNA dimer (1 µg) and each pooled siRNAs (50 nM) against various ESCRT factors were co-transfected into HepG2 cells. On day 5 post-transfection, no apparent cytotoxicity upon siRNA treatment was observed by the MTT assay. (B) The RNA expression of ESCRT factors can be successfully suppressed by siRNA treatment. Expression of VPS37C, MVB12A/B and CHMP4C, were not detectable in HepG2 cells. Data shown here are the averages of three independent experiments.
