# Supplementary material for: The Dual Role of an ESCRT-0 Component HGS in HBV Transcription and Naked Capsid Secretion
Source: PLoS Pathog. 2015 Oct 2;11(10):e1005123. doi: 10.1371/journal.ppat.1005123 (PMC4592276; doi:10.1371/journal.ppat.1005123)
Supplement: S2 Fig — (A) As an internal control for the transfection efficiencies in Fig 2E, a renilla luciferase reporter plasmid driven by a TK-promoter was included in the co-transfection experiment with the firefly luciferase reporter. No effect of si-HGS on the renilla activity was detected. Y-axis values represent the relative renilla luciferase activity in si-HGS-treated samples over the samples treated with non-targeting control siRNA. Data shown here are representative of at least three independent experiments. (B) Relative to the HBV tandem dimer replicon, treatment with si-HGS resulted in no appreciable effect on HBV replication by using a replicon plasmid pCHT-9/3091, whose transcription is driven by a CMV promoter. (DOCX) [file ppat.1005123.s002.docx]

**S2 Fig No effects of si-HGS on the reporter activity driven by a TK promoter and viral replication driven by a CMV promoter**

(A) As an internal control for the transfection efficiencies in Fig 2E, a renilla luciferase reporter plasmid driven by a TK-promoter was included in the co-transfection experiment with the firefly luciferase reporter. No effect of si-HGS on the renilla activity was detected. Y-axis values represent the relative renilla luciferase activity in si-HGS-treated samples over the samples treated with non-targeting control siRNA. Data shown here are representative of at least three independent experiments. (B) Relative to the HBV tandem dimer replicon, treatment with si-HGS resulted in no appreciable effect on HBV replication by using a replicon plasmid pCHT-9/3091, whose transcription is driven by a CMV promoter.
