# Supplementary material for: The Dual Role of an ESCRT-0 Component HGS in HBV Transcription and Naked Capsid Secretion
Source: PLoS Pathog. 2015 Oct 2;11(10):e1005123. doi: 10.1371/journal.ppat.1005123 (PMC4592276; doi:10.1371/journal.ppat.1005123)
Supplement: S3 Fig — Overexpression of HGS strongly reduced the levels of HBV DNA, RNA, and capsid particles. Plasmid DNAs of an HBV tandem dimer (ayw) and an HGS expression vector were co-transfected at a 2:1 (w/w) ratio into HepG2 and HuH-7 cells. On day 5 post-transfection, viral DNA synthesis, cytoplasmic viral RNA and intracellular capsid particles were examined by Southern, Northern, and native agarose gel electrophoresis. Similar to the CMV promoter (Fig 3A), the native HBV promoter of the tandem dimer plasmid is sensitive to the inhibitory effect of overexpressed HGS. (DOCX) [file ppat.1005123.s003.docx]

**S3 Fig Overexpression of HGS significantly suppressed viral transcription and replication driven by the HBV native promoter**

Overexpression of HGS strongly reduced the levels of HBV DNA, RNA, and capsid particles. Plasmid DNAs of an HBV tandem dimer (ayw) and an HGS expression vector were co-transfected at a 2:1 (w/w) ratio into HepG2 and HuH-7 cells. On day 5 post-transfection, viral DNA synthesis, cytoplasmic viral RNA and intracellular capsid particles were examined by Southern, Northern, and native agarose gel electrophoresis. Similar to the CMV promoter (Fig 3A), the native HBV promoter of the tandem dimer plasmid is sensitive to the inhibitory effect of overexpressed HGS.
