# Supplementary material for: The Dual Role of an ESCRT-0 Component HGS in HBV Transcription and Naked Capsid Secretion
Source: PLoS Pathog. 2015 Oct 2;11(10):e1005123. doi: 10.1371/journal.ppat.1005123 (PMC4592276; doi:10.1371/journal.ppat.1005123)
Supplement: S4 Fig — Six to eight week old BALB/c mice were tail-vein injected with 30 μg DNA of an HBV replicon pCHT-9/3091 and 6 μg DNA of an Flag-HGS expression vector. IHC analysis from serially sectioned liver showed that HBc (anti-HBc, Dako) and HGS (anti-Flag) proteins were highly co-expressed in the same hepatocytes at 1 dpi, as indicated by the red circles. (DOCX) [file ppat.1005123.s004.docx]

**S4 Fig Highly efficient co-expression of HBc and Flag-HGS proteins was detected by IHC in hepatocytes of serially sectioned liver in hydrodynamically injected mice**

Six to eight week old BALB/c mice were tail-vein injected with 30 µg DNA of an HBV replicon pCHT-9/3091 and 6 µg DNA of an Flag-HGS expression vector. IHC analysis from serially sectioned liver showed that HBc (anti-HBc, Dako) and HGS (anti-Flag) proteins were highly co-expressed in the same hepatocytes at 1 dpi, as indicated by the red circles.
