# Supplementary material for: The Dual Role of an ESCRT-0 Component HGS in HBV Transcription and Naked Capsid Secretion
Source: PLoS Pathog. 2015 Oct 2;11(10):e1005123. doi: 10.1371/journal.ppat.1005123 (PMC4592276; doi:10.1371/journal.ppat.1005123)
Supplement: S5 Fig — (A) HuH-7 cells were co-transfected with an HBV replicon pCHT-9/3091 and an HGS expression vector. On day 5 post-transfection, apoptosis was monitored by Western blot analysis of apoptotic markers (activated caspase 3 and PARP1 cleavage). (B) Cytotoxicity was measured by the lactate dehydrogenase (LDH) assay. No apparent apoptosis or cytotoxicity was observed. Data are representative of two independent experiments. (DOCX) [file ppat.1005123.s005.docx]

**S5 Fig Neither apoptosis nor cytotoxicity was detected in HGS-overexpressing cells**

(A) HuH-7 cells were co-transfected with an HBV replicon pCHT-9/3091 and an HGS expression vector. On day 5 post-transfection, apoptosis was monitored by Western blot analysis of apoptotic markers (activated caspase 3 and PARP1 cleavage). (B) Cytotoxicity was measured by the lactate dehydrogenase (LDH) assay. No apparent apoptosis or cytotoxicity was observed. Data are representative of two independent experiments.
