# Supplementary material for: The Dual Role of an ESCRT-0 Component HGS in HBV Transcription and Naked Capsid Secretion
Source: PLoS Pathog. 2015 Oct 2;11(10):e1005123. doi: 10.1371/journal.ppat.1005123 (PMC4592276; doi:10.1371/journal.ppat.1005123)
Supplement: S6 Fig — The ratios of banding intensities among various viral and subviral particles were quantified by Image J analysis. An estimate for the differential effects of HGS is summarized below: naked capsids: empty virions: HBsAg particles: complete virions = 1.5X increase: 1.6X decrease: 2.5X decrease: 3X decrease. Empty virions were calculated according to the data from polymerase mutants (Y63D and 2310), while complete virions were based on the Southern blot data using native agarose gel assay (Fig 6). (DOCX) [file ppat.1005123.s006.docx]

**S6 Fig Quantitative comparisons of the effect of overexpressed HGS on various viral and subviral particles in HGS-transfected HuH-7 cells**

The ratios of banding intensities among various viral and subviral particles were quantified by Image J analysis. An estimate for the differential effects of HGS is summarized below: naked capsids: empty virions: HBsAg particles: complete virions= 1.5X increase: 1.6X decrease: 2.5X decrease: 3X decrease. Empty virions were calculated according to the data from polymerase mutants (Y63D and 2310), while complete virions were based on the Southern blot data using native agarose gel assay (Fig 6).
