# Supplementary material for: The Dual Role of an ESCRT-0 Component HGS in HBV Transcription and Naked Capsid Secretion
Source: PLoS Pathog. 2015 Oct 2;11(10):e1005123. doi: 10.1371/journal.ppat.1005123 (PMC4592276; doi:10.1371/journal.ppat.1005123)
Supplement: S7 Fig — (A) HGS dUIM maintained the suppression effect on viral replication, albeit its potency appeared to be weaker than that of the wild type HGS. HepG2 cells were co-transfected with HBV dimer and HGS expression vectors at a 2:1 (w/w) ratio. Five days post-transfection, viral DNAs were harvested and compared by Southern blot analysis. (B) Overexpression of wild type or mutant HGS inhibited HBV EnhII-Cp activity. The plasmid of pGL3-EnhII-Cp (as depicted in Fig 2E), control plasmid pRL-TK, and HGS expression vectors, were co-transfected into HepG2 and HuH-7 cells. Two days post-transfection, cell lysates were subjected to Western blot and reporter analysis. (DOCX) [file ppat.1005123.s007.docx]

**S7 Fig UIM domain-truncated HGS (HGS dUIM) exhibited a reduced potency in suppressing HBV transcription and viral replication**

(A) HGS dUIM maintained the suppression effect on viral replication, albeit its potency appeared to be weaker than that of the wild type HGS. HepG2 cells were co-transfected with HBV dimer and HGS expression vectors at a 2:1 (w/w) ratio. Five days post-transfection, viral DNAs were harvested and compared by Southern blot analysis. (B) Overexpression of wild type or mutant HGS inhibited HBV EnhII-Cp activity. The plasmid of pGL3-EnhII-Cp (as depicted in Fig 2E), control plasmid pRL-TK, and HGS expression vectors, were co-transfected into HepG2 and HuH-7 cells. Two days post-transfection, cell lysates were subjected to Western blot and reporter analysis.
