# Supplementary material for: The Dual Role of an ESCRT-0 Component HGS in HBV Transcription and Naked Capsid Secretion
Source: PLoS Pathog. 2015 Oct 2;11(10):e1005123. doi: 10.1371/journal.ppat.1005123 (PMC4592276; doi:10.1371/journal.ppat.1005123)
Supplement: S8 Fig — (A) An HBV replicon pCHT-9/3091 was transfected into HepG2 cells at 48 hours before IFA. Endogenous HGS showed a punctate distribution of weak signal intensity and was sparsely co-localized with the cytoplasmic HBc (anti-HBc, Hyb-3120). (B) The majority of Flag-HGS were clustering into an enlarged punctate structure in the cytoplasm, which partially co-localized with an endosome marker EEA-1. (DOCX) [file ppat.1005123.s008.docx]

**S8 Fig The localization patterns of endogenous and exogenous HGS were examined in HepG2 cells**

(A) An HBV replicon pCHT-9/3091 was transfected into HepG2 cells at 48 hours before IFA. Endogenous HGS showed a punctate distribution of weak signal intensity and was sparsely co-localized with the cytoplasmic HBc (anti-HBc, Hyb-3120). (B) The majority of Flag-HGS were clustering into an enlarged punctate structure in the cytoplasm, which partially co-localized with an endosome marker EEA-1.
