# Supplementary material for: The Dual Role of an ESCRT-0 Component HGS in HBV Transcription and Naked Capsid Secretion
Source: PLoS Pathog. 2015 Oct 2;11(10):e1005123. doi: 10.1371/journal.ppat.1005123 (PMC4592276; doi:10.1371/journal.ppat.1005123)
Supplement: S9 Fig — (DOCX) [file ppat.1005123.s009.docx]

**S9 Fig Both punctate (white arrow) and peripheral (black arrow) localizations of the exogenous HGS protein can be observed by IHC in sectioned livers of BALB/c mice hydrodynamically injected with an HGS-expression vector**
